# Plant growth-promoting rhizobacteria promote plant size inequality

**DOI:** 10.1038/s41598-018-32111-z

**Published:** 2018-09-14

**Authors:** Alan C. Gange, Kiran R. Gadhave

**Affiliations:** 1 School of Biological Sciences, Royal Holloway, University of London, Egham, Surrey TW20 0EX UK; 2 Present Address: Department of Entomology and Plant Pathology, North Carolina State University, Raleigh, NC 27606 USA

## Abstract

The uniformity of crop yield is extremely important for consumers and of as much relevance to the grower as overall yield. However, size inequality within a plant population is rarely measured and has never before been considered in relation to the use of beneficial microbes for yield enhancement. For the first time, we show that addition of soil bacteria to calabrese plants significantly increased size inequality. These effects were usually more apparent in above-ground biomass. This was caused by some (but not all) plants growing very large when inoculated with bacteria, while control plants were mostly small. We suggest that the main reason is the incompatibility of the inoculated bacteria with those already present in the rhizosphere. In some cases the inoculum matched the indigenous community, providing a benefit to plant growth, while often it did not and plants remained relatively small. We conclude that analyses of size inequality should be an integral part of experiments using microbial soil amendments. These analyses can help to inform the production of more effective microbial products and to ensure that the integration of beneficial microbes into sustainable production systems does not impair uniformity in yield.

## Introduction

Conventional agriculture, through the use of selected varieties and inputs of fertilizers and pesticides, seeks to maximise not only total yield, but also the uniformity of the crop. Much research has shown that uniformity of size and appearance of a fruit or vegetable influences consumer choice^1^. Indeed, uniformity of size is a more important factor than knowledge of pesticide application to the crop, and even scent and flavour^2,3^. Furthermore, in field crops such as calabrese, a lack of uniformity in plant size makes harvesting difficult and prolonged, thereby increasing production costs^4^.

Organic production systems, designed to minimise the impact of agriculture on humans and the environment, have seen a rise in popularity over the last 20 years. While the environmental benefits cannot be disputed, the main disadvantage is the difference in total yield, which may be 20–30% less than that of conventional systems^5,6^. However, an unrecognised aspect of organic agriculture may be an increase in size inequality (i.e. a decrease in uniformity) of the crop. Levels of beneficial soil microbes, such as arbuscular mycorrhizal (AM) fungi, and foliar-feeding insects and pathogenic fungi may all be higher in organic systems and all have the potential to increase size inequality^7–9^. This is because not all plants in a population are colonized to the same degree by AM fungi, or attacked equally by insects or pathogens, resulting in plant size distributions that become ‘stretched’ at the lower end (many small individuals) and/or at the upper end (fewer very large individuals that escape attack or are mycorrhizal).

An important aspect of any agricultural system is the quality of the soil, with much recent interest focused on improving quality, and ultimately yield, through the addition of microbial inoculants^10^. Prominent amongst these inoculants are plant growth-promoting rhizobacteria (PGPR), including species in genera such as *Azotobacter*, *Bacillus*, and *Pseudomonas*^11^. These PGPR can enhance plant growth through nutrient recycling, nitrogen fixation, phytohormone production, solubilisation of nutrients such as P, K and Fe, and enhancing plant resistance to pests and diseases^11^. Therefore, the primary aim of PGPR addition is to increase overall yield. However, whether their addition has any effects on crop uniformity is unknown. Thus, the aim of this paper is to examine the effects of PGPR addition on size inequality within an important field crop, *Brassica oleracea* var. *italica* (calabrese). Our holistic approach to studying the effects of ubiquitous PGPR (*Bacillus* spp.) on calabrese growth, endophytic bacterial community and plant biotic stress in previous studies led us to investigate *Bacillus*-mediated effects on plant size inequality. In a series of experiments in controlled and field conditions, with single and multiple *Bacillus* spp. additions, we showed that these PGPR affected plant growth^12^, altered endophytic bacterial community diversity, evenness and composition^13^, and suppressed cabbage aphid growth^14^ and field incidence^15^ in a context specific manner.

Calabrese is sensitive to variation in soil N and water availability, which can often lead to a lack of uniformity in the crop^16^. Therefore, it is relevant that nutrient delivery to roots and amelioration of drought stress are just two of the benefits that PGPR can provide to plants^11^. However, much attention has been focused on the inconsistent efficacy of PGPR inoculants in field conditions, due mainly to compatibility issues of species in the inoculant with those in the rhizosphere and the heterogeneous distribution of nutrients in soils^17,18^. We therefore hypothesized that addition of PGPR to the roots of calabrese plants would increase plant size inequality, due to the differential ability of bacterial species to establish in the rhizosphere^19^. Furthermore, we hypothesized that plant size inequality would be further amplified in field conditions and with multispecies bacterial inoculants, because of nutrient heterogeneity and competitive interactions between the bacterial species^13,18^.

## Results and Discussion

**Figure 1:**
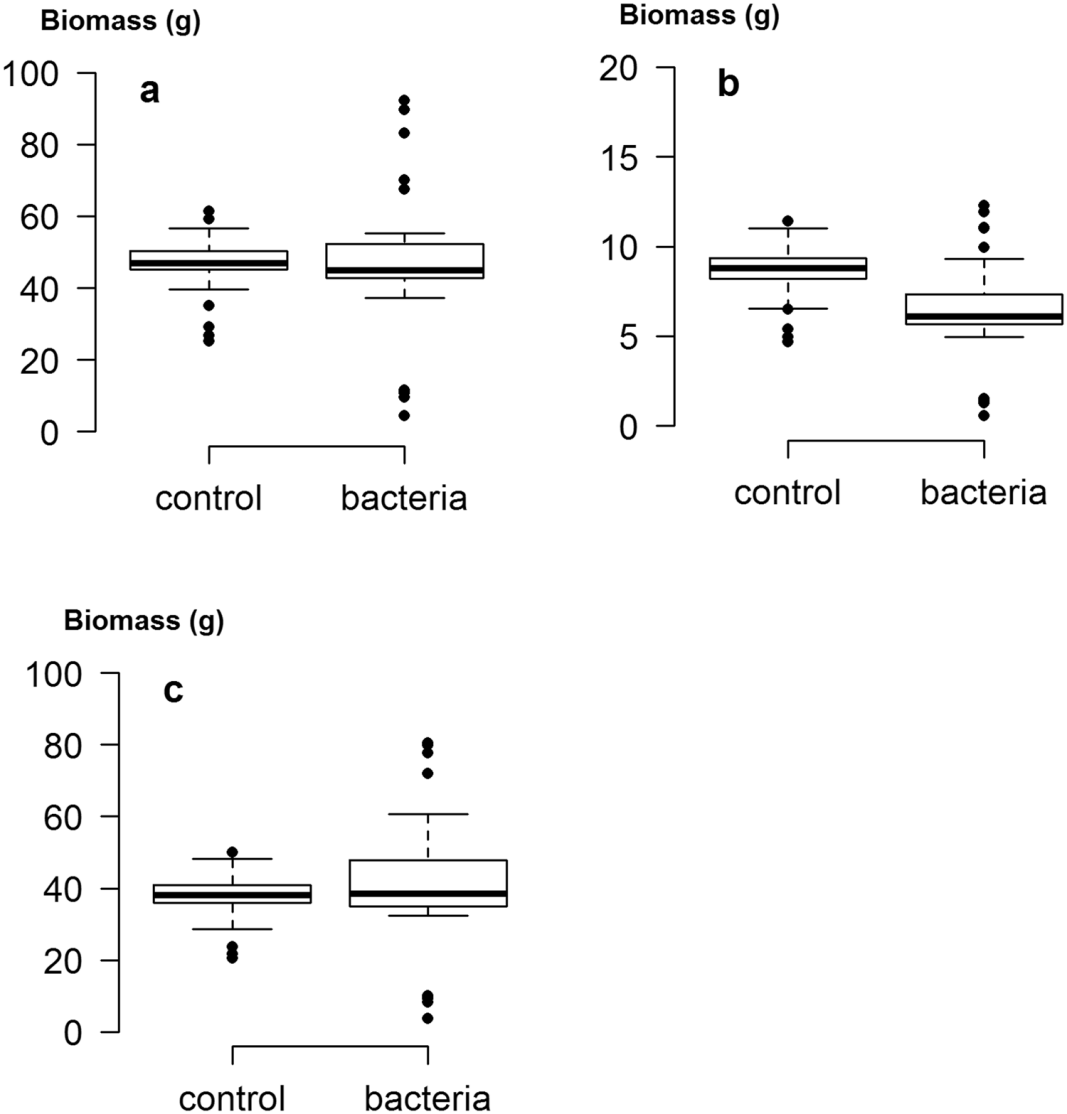
Box plots showing the range in size distributions for (**a**) total biomass, (**b**) root biomass and (**c**) shoot biomass of calabrese plants grown in the controlled experiment, with and without the addition of a mixture of plant growth-promoting rhizobacteria. The horizontal line within the box is the median, while edges of the box represent the inter-quartile ranges. The whiskers depict 1.5x the inter-quartile ranges, while points depict outliers beyond the whiskers.

**Figure 2:**
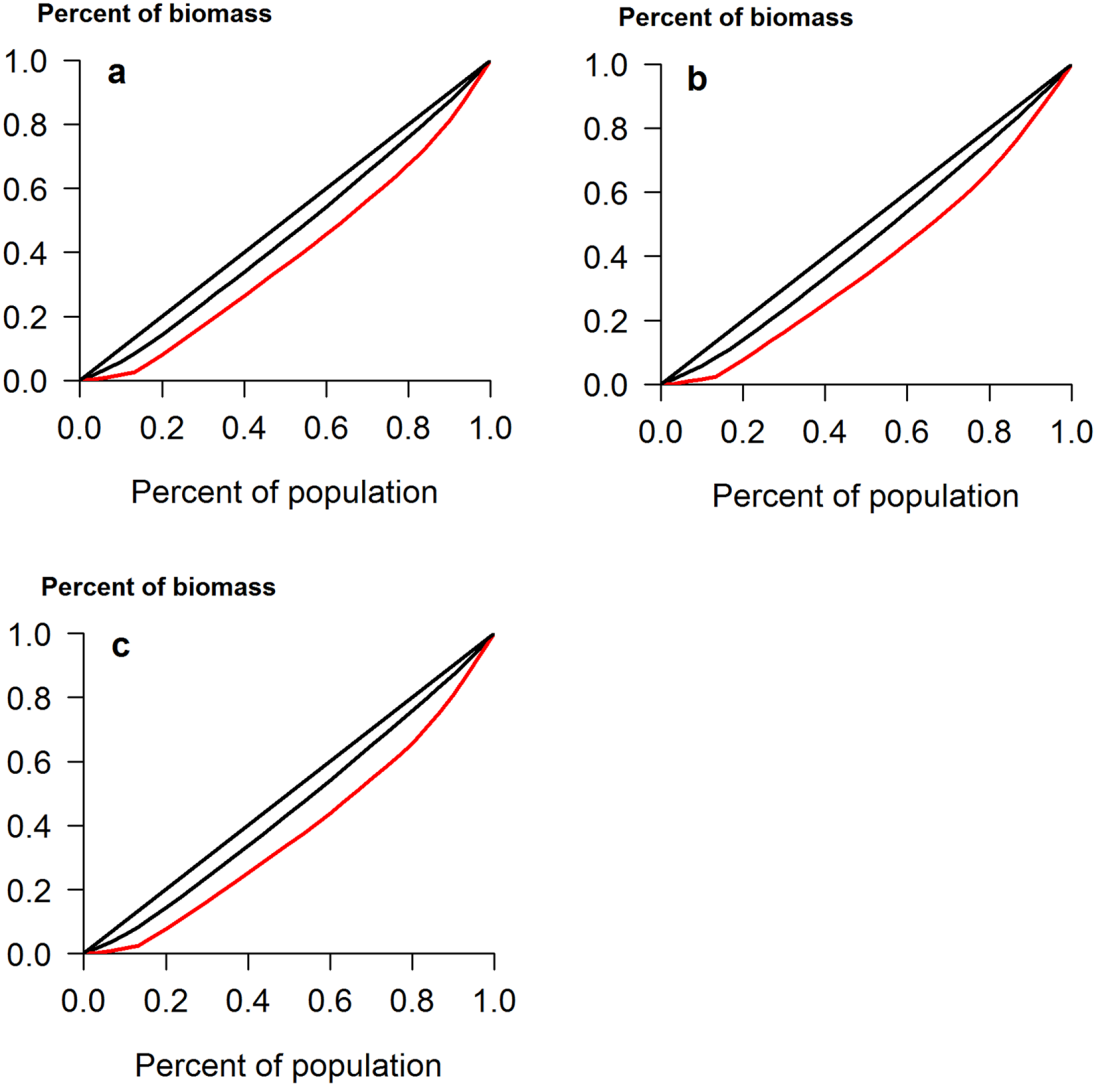
Lorenz curves for (**a**) total biomass, (**b**) root biomass and (**c**) shoot biomass of calabrese plants grown in the controlled experiment. The diagonal straight line represents the line of equality. Control plants depicted in black, PGPR addition in red.

Addition of the bacterial mixture only had an effect on root biomass in the controlled experiment, which was reduced when PGPR were applied (Fig. 1). However, bacterial addition caused a significant increase in the inequality of total biomass, raising the CV from 18% to 44% (*Z* = 4.1, *p* < 0.001) (Table 1). Similar significant results were seen with both root and shoot biomass and were reflected in the Gini coefficient and the Gini Mean of Differences (Table 1). A notable feature of the Lorenz asymmetry coefficients was that those for control plants were always less than one, while those for treated plants were all greater than one (Fig. 2 and Table 1). The former indicates that the majority of control plants were small, while the latter indicates that some large individuals occurred when bacteria were applied. PGPR addition therefore increased plant size variation, upholding our original hypothesis.

**Table 1:** Measures of inequality for calabrese plants grown in controlled conditions, with and without the addition of PGPR.

	Total biomass	Root biomass	Shoot biomass
CV control	18.03	18.7	18.1
CV bacteria	**44**.**01**	**45**.**09**	**46**.**52**
Z test	4.13, *p* < 0.001	4.05, *p* < 0.001	4.31, *p* < 0.001
Gini control (95% CI)	0.0952 (0.0645–0.1432)	0.101 (0.0699–0.1466)	0.0968 (0.0659–0.1422)
Gini bacteria (95% CI)	**0**.**231** (0.1582–0.3468)	**0**.**247** (0.1777–0.3565)	**0**.**251** (0.1784–0.3646)
Gini MD control (95% CI)	8.789 (6.097–12.603)	1.728 (1.221–2.376)	7.252 (5.084–10.203)
Gini MD bacteria (95% CI)	**21**.**699** (14.74–30.55)	3.223 (2.36–4.283)	**20**.**906** (14.62–28.29)
Lorenz AC control	0.8774	0.7703	0.8537
Lorenz AC bacteria	1.0918	1.0146	1.0587

CV is Coefficient of Variation, Gini is Gini Coefficient, Gini MD is Gini Mean of Differences and Lorenz AC is Lorenz Asymmetry Coefficient. Differences between bacterial treatments and the control are indicated in bold text. Differences in Gini Coefficients and Gini MD determined by non-overlap of confidence intervals at *p* = 0.05.

There are many studies in which bacterial addition has been shown to increase plant biomass^11^, but all previous authors report changes in mean plant size, rather than inequality. Patchy effects of inoculation have been recorded before^17^, but only at the overall treatment or plot level and never at the within-treatment level, as described here. The most likely reason for the effects seen is variable colonization and establishment of the PGPR in the rhizosphere^19^. The addition of just one bacterial species can have major effects on the structure of the rhizosphere microbial community, depending on whether the added species was already a member of that community or not^13^. Such effects can be magnified if a mixture of PGPR is added^18^. Thus, the most plausible explanation is that the mixed inoculum of bacteria did not establish to the same degree in each pot. When successful rhizosphere colonization occurred, plants benefited from the addition and grew large, but in some individuals, growth promotion did not occur, leading to the increase in inequality and decrease in uniformity of plant size. Commercial potting media may vary from bag to bag in their physical and chemical characteristics^20^ and it is highly likely that they also vary in soil microbial community composition too.

**Figure 3:**
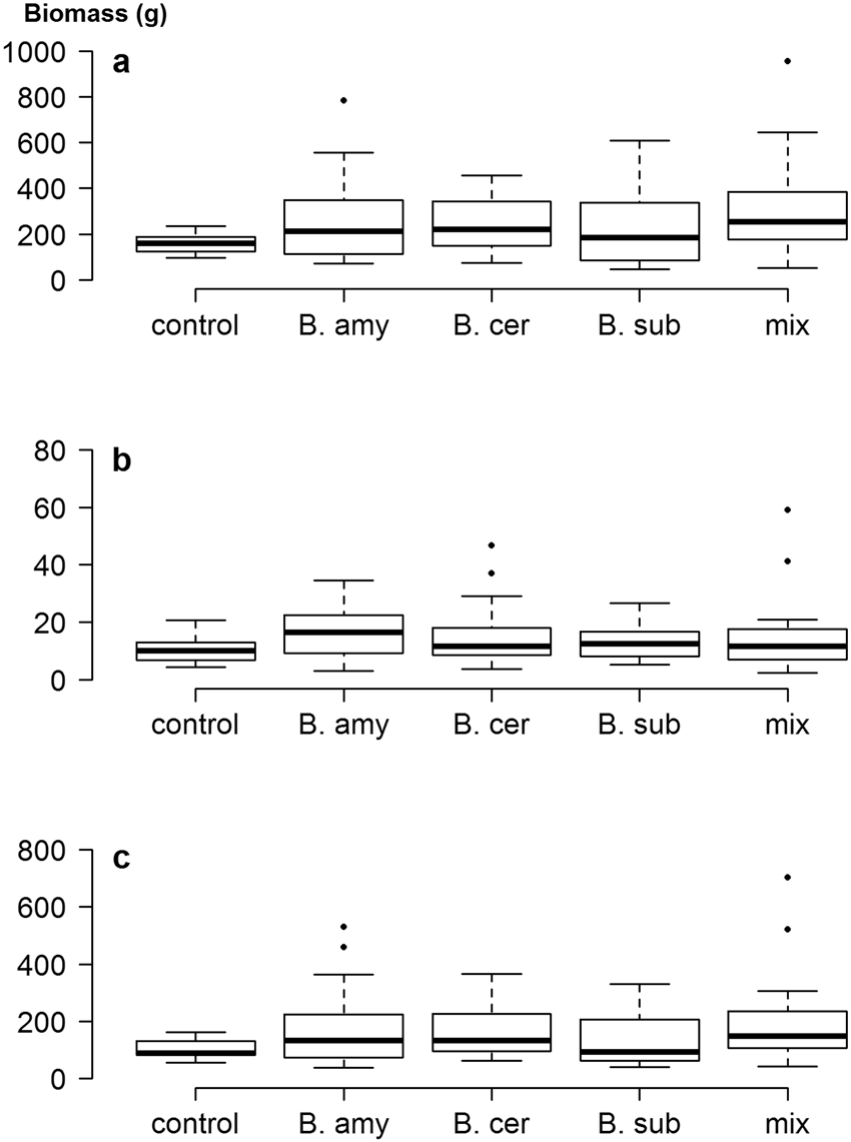
Box plots showing the range in size distributions for (**a**) total biomass, (**b**) root biomass and (**c**) shoot biomass of calabrese plants grown in UK field soil, with and without the addition of plant growth-promoting rhizobacteria. Addition of PGPR indicated by: B. amy: *Bacillus amyloliquefaciens*; B. cer: *B*. *cereus*; B. sub: *B*. *subtilis*; mix: mixture of all three species.

**Figure 4:**
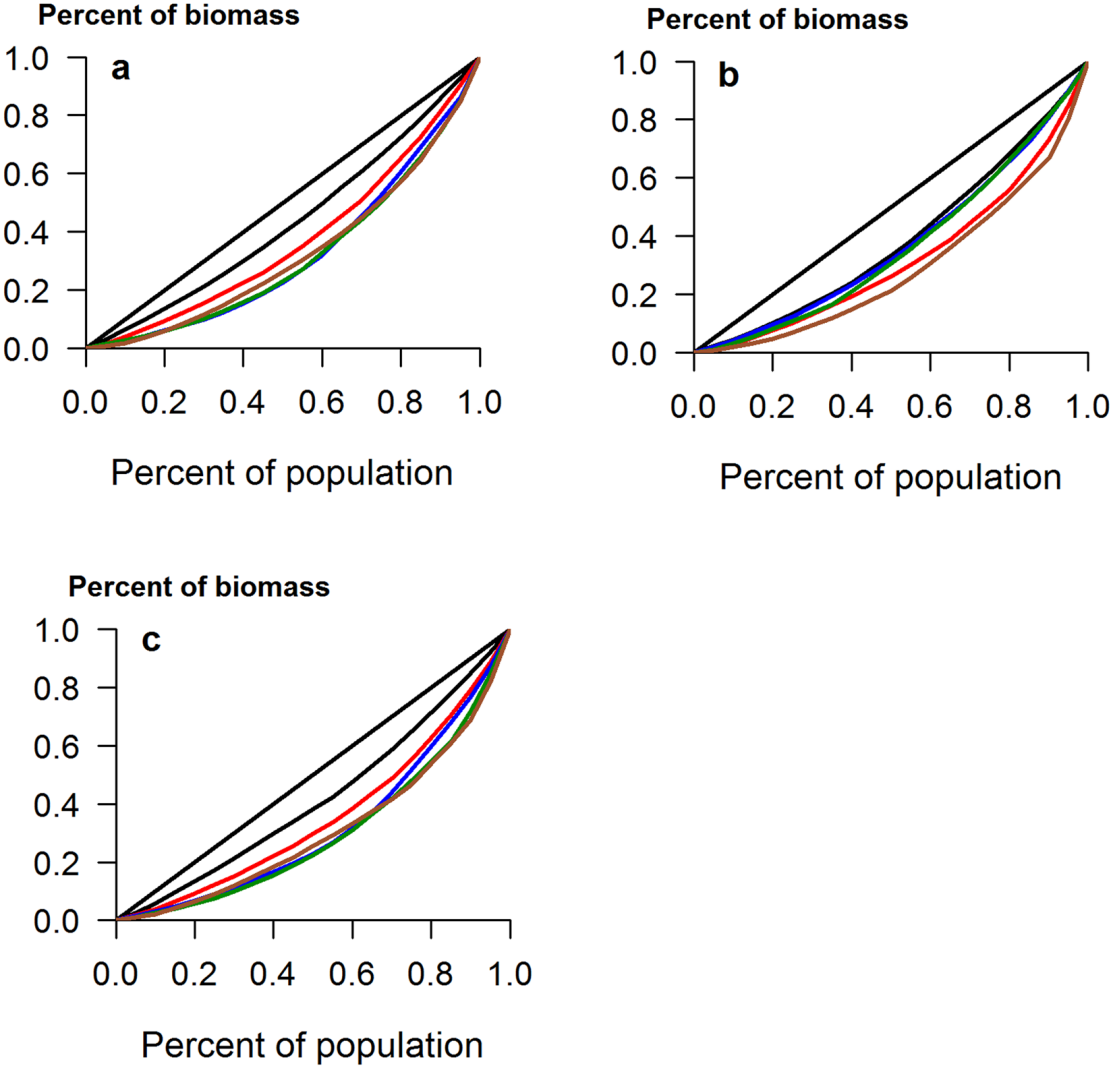
Lorenz curves for (**a**) total biomass, (**b**) root biomass and (**c**) shoot biomass of calabrese plants grown in UK field soil. The diagonal straight line represents the line of equality. Control plants depicted in black, *B*. *amyloliquefaciens* addition in green, *B*. *cereus* addition in red, *B*. *subtilis* addition in blue and the mixed inoculum in brown.

Field populations of soil bacteria are notoriously heterogeneous at all spatial scales^21^ and so it is not surprising that the CVs for field grown plants in the UK were considerably larger than those for the pot-grown plants (Table 2). It should be noted that these plants were also subject to insect attack, which can affect size inequality^7^. Here, addition of either a single inoculation or a mixture of species increased the CV of total biomass from 25.5% in controls up to 71% in plants inoculated with *B*. *amyloliquefaciens* (*Z* = 3.4, *p* < 0.001). It is probably no coincidence that addition of this bacterium also caused the greatest changes in the indigenous rhizosphere microbial community in this experiment^13^. There was a much greater range in biomass in all treated plants, relative to controls (Fig. 3), and the Lorenz curves for total and shoot biomass showed clear increases in inequality when any PGPR were applied (Fig. 4). However, addition of *B*. *subtilis* or *B*. *amyloliquefaciens* had no effect on the inequality of root biomass (Table 2 and Fig. 4b), showing that effects of PGPR addition are not just species-specific, but also plant-organ specific. Furthermore, the addition of inoculants did not result in increases in the Lorenz asymmetry coefficients (Table 2) and in all cases, populations were composed of a majority of small individuals. This is likely due to the incompatibility of the added species with those already in the rhizosphere, suggesting that relatively few plants benefited from the addition of PGPR. Indeed, no significant increases in mean plant size were found in this study, due in part to the large inequality seen in some treatments^15^.

**Table 2:** Measures of inequality for calabrese plants grown in UK and Indian field conditions, with and without the addition of PGPR. Abbreviations as in Table 1.

	UK field experiment			Indian field experiment		
	Total biomass	Root biomass	Shoot biomass	Total biomass	Root biomass	Shoot biomass
CV control	25.51	41.25	29.14	23.36	38.22	21.36
CV *B*. *amyloliquefaciens* Z test	**71**.**41** 3.41, *p* < 0.001	48.83 0.619, NS	**77**.**61** 3.16, *p* < 0.01	**42**.**45** 2.29, *p* < 0.05	41.86 0.34, NS	**42**.**78** 2.65, *p* < 0.01
CV *B*. *cereus* Z test	**48**.**19** 2.37, *p* < 0.05	**70**.**79** 2.82, *p* < 0.05	**53**.**33** 2.21, *p* < 0.05	38.17 1.91, NS	41.53 0.31, NS	**37**.**91** 2.24, *p* < 0.05
CV *B*. *subtilis* Z test	**67**.**42** 3.29, *p* < 0.001	46.09 0.41, NS	**66**.**83** 2.83, *p* < 0.01	**41**.**79** 2.24, *p* < 0.05	43.69 0.51, NS	**41**.**79** 2.57, *p* < 0.05
CV mixture Z test	**70**.**01** 3.36, *p* < 0.001	**90**.**15** 2.37, *p* < 0.05	**82**.**68** 3.27, *p* < 0.01	**39**.**57** 2.06, *p* < 0.05	35.93 0.24, NS	**40**.**11** 2.43, *p* < 0.05
Gini control (95% CI)	0.150 (0.123–0.189)	0.237 (0.187–0.312)	0.167 (0.140–0.215)	0.128 (0.092–0.188)	0.221 (0.176–0.286)	0.122 (0.092–0.168)
Gini *B*. *amyloliquefaciens* (95% CI)	**0**.**391** (0.317–0.490)	0.282 (0.219–0.331)	**0**.**413** (0.332–0.507)	**0**.**249** (0.194–0.301)	0.244 (0.186–0.311)	**0**.**251** (0.195–0.313)
Gini *B*. *cereus* (95% CI)	**0**.**277** (0.234–0.352)	0.367 (0.290–0.471)	**0**.**299** (0.254–0.374)	0.214 (0.167–0.286)	0.233 (0.181–0.328)	0.212 (0.168–0.279)
Gini *B*. *subtilis* (95% CI)	**0**.**379** (0.307–0.476)	0.263 (0.209–0.331)	**0**.**375** (0.323–0.444)	**0**.**243** (0.195–0.318)	0.253 (0.205–0.319)	**0**.**243** (0.185–0.318)
Gini mixture (95% CI)	**0**.**372** (0.287–0.493)	**0**.**437** (0.317–0.567)	**0**.**404** (0.293–0.523)	0.230 (0.170–0.313)	0.206 (0.143–0.295)	**0**.**233** (0.174–0.316)
Gini MD control (95% CI)	48.25 (39.59–60.11)	4.86 (3.86–6.94)	35.04 (27.92–45.3)	34.42 (24.66–55.71)	4.83 (3.75–6.44)	30.16 (22.60–42.44)
Gini MD *B*. *amyloliquefaciens* (95% CI)	**204**.**45** (148.3–319.7)	**9**.**35** (7.39–12.86)	**145**.**22** (95.2–215.9)	**99**.**69** (84.56–115.38)	**8**.**13** (6.52–10.19)	**92**.**05** (78.17–106.30)
Gini MD *B*. *cereus* (95% CI)	**133**.**07** (105.1–166.9)	**11**.**49** (7.37–18.01)	**98**.**53** (72.65–132.52)	56.11 (40.15–74.32)	5.37 (3.84–7.48)	50.83 (35.86–67.33)
Gini MD *B*. *subtilis* (95% CI)	**168**.**24** (133.52–252.8)	**6**.**92** (5.22–9.28)	**98**.**97** (77.32–130.97)	**90**.**29** (72.82–116.56)	7.40 (6.18–9.66)	**83**.**13** (66.75–107.81)
Gini MD mixture (95% CI)	**234**.**01** (157.3–370.8)	**13**.**17** (7.36–23.66)	**156**.**80** (87.6–268.2)	**105**.**14** (83.60–134.20)	7.50 (5.63–9.89)	**98**.**05** (77.80–125.09)
LAC control	0.998	0.928	1.073	1.208	0.923	1.161
LAC *B*. *amyloliquefaciens*	0.880	0.859	0.976	0.802	0.867	0.804
LAC *B*. *cereus*	0.976	1.084	1.049	1.256	1.121	1.254
LAC *B*. *subtilis*	0.927	0.945	0.969	0.738	0.853	0.717
LAC mixture	1.078	0.913	1.218	0.986	0.734	1.015

Differences between bacterial treatments and the control are indicated in bold text. Differences in Gini Coefficients and Gini MD determined by non-overlap of confidence intervals at *p* = 0.05; NS = no significant difference.

**Figure 5:**
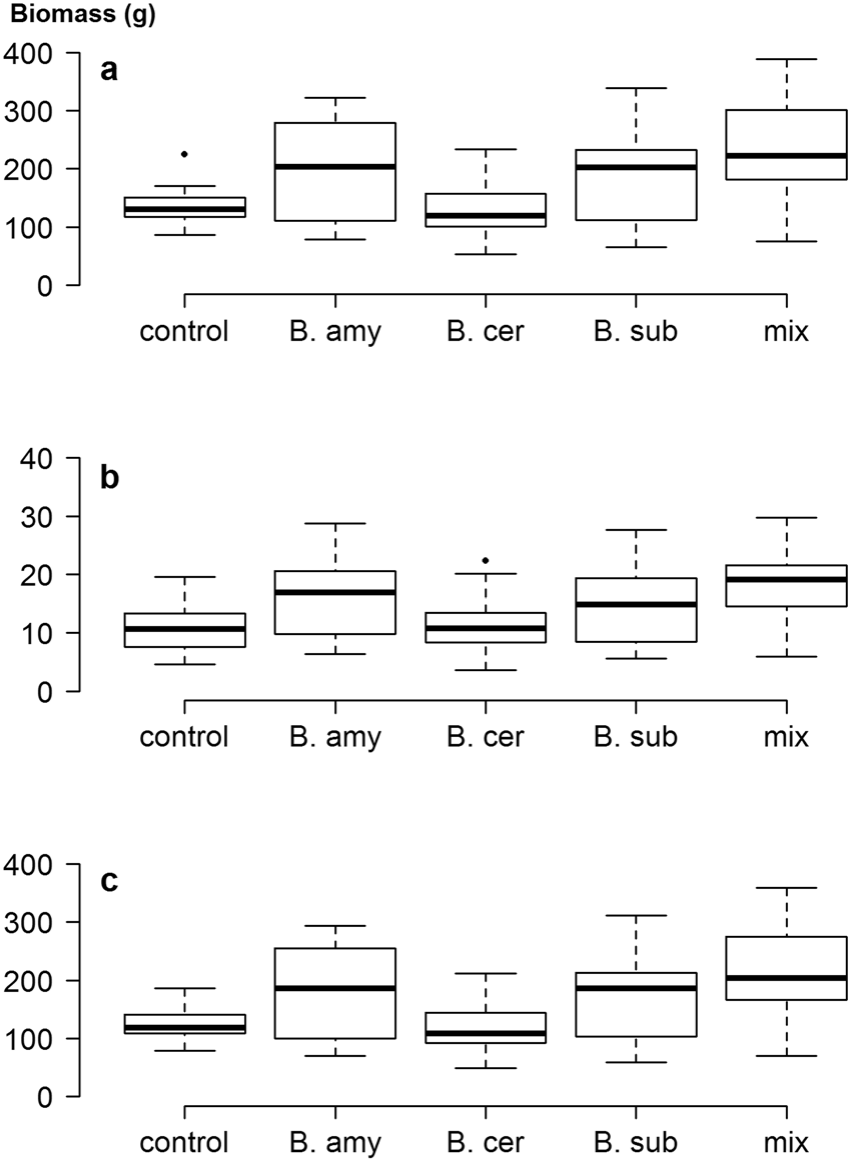
Box plots showing the range in size distributions for (**a**) total biomass, (**b**) root biomass and (**c**) shoot biomass of calabrese plants grown in Indian field soil, with and without the addition of plant growth-promoting rhizobacteria.

**Figure 6:**
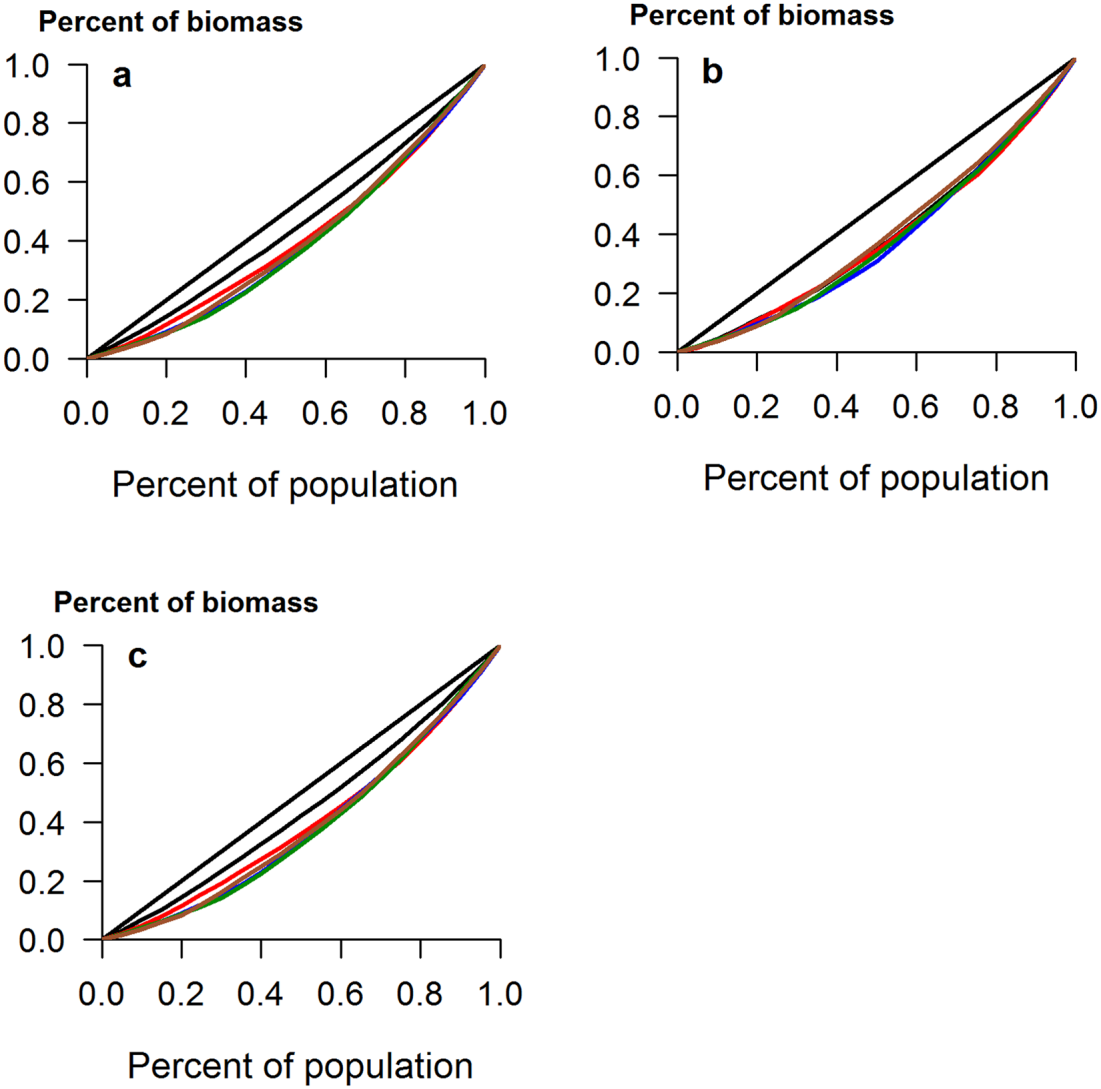
Lorenz curves for (**a**) total biomass, (**b**) root biomass and (**c**) shoot biomass of calabrese plants grown in Indian field soil. Legend as in Fig. 4.

Plants grew more rapidly in the warmer climate of India, but were smaller in size at harvest than those from the UK. Addition of the mixture increased plant biomass (Fig. 5) and the CV for total biomass of controls was similar to that in the UK, at 23.4% (Table 2). Most PGPR treatments increased size inequality, although *B*. *cereus* had far fewer effects than in the UK (Fig. 5). Addition of *B*. *amyloliquefaciens* again produced the greatest increase in the CV of total biomass, raising this to 42.4% (*Z* = 2.29, *p* < 0.05). As with UK plants, addition of single species and the mixture caused increases in the Gini coefficient and Gini Mean of Differences, with greatest increases seen when *B*. *amyloliquefaciens* or the mixture were applied (Table 2). As with the UK experiment, inequality of root biomass was much less affected than shoot biomass (Table 2). Lorenz asymmetry coefficients also showed a similar trend to the UK, in that the majority of plants in the bacterial treatments were small. Coefficients in *B*. *cereus* treatments were very similar to controls, reinforcing the conclusion that addition of this species had no measurable effects on the plants (Fig. 6).

Despite the differences in calabrese cultivars and the strains of *Bacillus* spp. used in the two field experiments, the results were consistent in that PGPR addition increased inequality, but also showed context-specificity, as inoculation with the same bacteria did not produce the same effect in each place. The most likely explanation is that the natural rhizosphere bacterial communities in the UK and India differed^21^, resulting in inconsistent effects. It has been suggested that inoculation of seed with PGPR can help to overcome such problems and enable the added bacteria to establish better, in the face of antagonism from indigenous species^22^. However, we inoculated seeds in our field experiments and still found patchy effects of inoculation, even though the inoculated species successfully colonized the rhizosphere^13^. Perhaps of more promise is the encapsulation of bacterial cells within products such as sodium alginate^23^, though these authors also acknowledge that the most appropriate encapsulation method likely varies from one bacterial species to another. Intriguingly, in a study that involved one of the species used here (*B*. *subtilis*), addition of encapsulated bacteria reduced inequality of lettuce growth, relative to controls^24^.

The analysis of heterogeneity within crops is of much interest from the economic point of view, as consumers are willing to pay a premium for attributes that include quality and uniformity^25^. Harvesting of crops such as calabrese is more efficient and economical when uniformity of the product is high^4^. The size of an individual calabrese plant may be limited by the availability of N and water, both of which PGPR could potentially help to ameliorate. However, our results clearly demonstrate the need for a better understanding of how individual plants respond to inoculation, perhaps with tailoring of products to specific soil types to account for differences in indigenous microbes and soil nutrients^10^. Furthermore, they also have critical importance for identification of the limiting abiotic factors that may restrict yield^26^. In any manipulative experiment, secondary statistics of size hierarchies, or size inequality, are useful as measures of plant competitive ability and for understanding how abiotic factors such as water or fertilization affect plant growth^26,27^. If agriculture is to become more sustainable, with less reliance on synthetic pesticides and inorganic fertilizers, then microbes such as PGPR will play a crucial role^11^. In particular, PGPR offer the potential to reduce the yield gap between conventional and organic systems^5^. We suggest that in future studies of organic systems, an analysis of size inequality should accompany experiments involving PGPR and plant growth, to inform inoculation methods, and ensure that overall yield enhancements are not at the expense of crop quality.

## Conclusions

We have presented the first demonstration that application of PGPR may promote size inequality within crop yield, even if there are no absolute changes in yield. These results have important implications for consumers as well as farmers and growers and those who produce microbial inoculants.

Uniformity within a crop strongly influences consumer preference and if PGPR are to be integrated into organic production systems, there is a need to ensure that overall quality of the product is not reduced, even if yield is enhanced. Any attempt to reduce the yield gap between conventional and organic systems should involve an analysis of size inequality, to ensure marketability is maintained.

Analyses of size inequality can also tell us a lot about the compatibility of products with the rhizosphere. Our results are most likely caused by inconsistent establishment of the inoculated species in soil, meaning that some plants benefited from inoculation and grew large, while many did not and remained small. This situation is the opposite of the uniform distribution that growers aim to produce. Future PGPR products need to be more tailored to specific crop situations or soil types, and a universal inoculant is therefore unlikely to be successful at present.

## Materials and Methods

### PGPR preparation

All experiments formed part of a three-year study into the effects of PGPR on calabrese growth, and the insect community associated with the plants, and are described in full therein^12^. Three experiments, one in controlled conditions and two in field plots (one in the UK and one in India) were conducted. All used *B*. *oleracea* var. *italica* and all involved single inoculations of *Bacillus amyloliquefaciens* subsp. *plantarum*, *B*. *subtilis* and *B*. *cereus*. In the controlled experiment and UK field experiment, strains of *B*. *amyloliquefaciens* FZB42BGSC10A6, *B*. *subtilis* NRRLB23051 and *B*. *cereus* No. 8 FW Athal or a mixed inoculum of all three species, were used, and compared with controls. All bacterial strains were obtained from Dr B. Raymond and were originally isolated from *Arabidopsis thaliana*, (Brassicaceae) in Egham, UK, and screened initially for their ability to colonize calabrese in laboratory and field conditions^12^.

In the Indian field experiment, strains of *B*. *cereus* 2028 (MTCC Accession No. 9017), *B*. *subtilis* N11 (MTCC Accession No. 8141) and *B*. *amyloliquefaciens* TFRI4 (MTCC Accession No. 10439) obtained from the Microbial Type Culture Collection and Gene Bank (MTCC), Chandigarh, India were used. All these *Bacillus* spp. have been previously reported to be indigenous in India and to colonize an array of crops as endophytes^28^. Details of the origin of each strain can be found at https://mtccindia.res.in/catalog using the MTCC Accession numbers specified above.

Bacterial strains for all experiments were preserved at −80 °C in 80% glycerol and recovered in 20 ml lysogeny broth (LB) overnight on a rotary shaker at 37 °C. Each culture was then diluted to 10^−5^ in 0.85% saline water and 50 µl of each diluted culture were spread on 9 cm diameter petri plates containing LB agar medium to determine the viable population count. Single inoculations consisted of 240 ml of a 10^8^ cfu/mL suspension of each species in 0.85% saline water, while the mixed inoculum consisted of 80 ml of each species. Control plants received 240 ml of sterilized distilled water. As a standard practice, 0.85% saline water was used for bacterial dilution as it is known to act as an isotonic solution minimising differences in osmotic pressure inside and outside of bacterial cells. Given the nature and frequency of bacterial inoculant application, it is unlikely that the use of sterilized distilled water instead of 0.85% saline water in control plants would have impacted the plant biomass.

### Controlled experiment

Calabrese seeds (cv. Green Sprouting (Country Value Seeds, UK)) were surface sterilized by immersion in a 2% solution of sodium hypochlorite for 20 min. Seeds were then washed five times in sterile distilled water and decanted to a sterile petri dish. Seeds were transferred to each of two sterile plates containing reduced strength Murashige and Skoog seed germination medium. Plates were placed in the dark at 17 °C for 10 d, after which time two vigorous seedlings were each transplanted into 1 L pots containing 800 ml of John Innes Number 3 (soil based compost) (Westland Horticulture Ltd, Huntingdon, UK). There were 30 replicates of each of two treatments; control plants with 240 ml of sterilized distilled water and bacterial inoculation with a 240 ml mixture of the three bacterial species.

Bacterial inoculations were applied immediately after transplanting and after a further 7d, the weaker seedling was removed. Plants were then grown for 12 w in constant conditions of 17 °C and 16:8 L:D and irrigated twice a week with sterilized distilled water. They were then harvested and fresh and dry root and shoot biomass recorded.

### UK field experiment

This field experiment took place in Egham, Surrey, UK (51.4247°N, 0.5669°W) from June to October 2013 and represents the plant data from a study of PGPR and insect communities^15^, where a full description can be found. The experimental plot had a free-draining slightly acid loamy soil (pH 5.4), with low fertility. Briefly, calabrese seeds (same variety as above) were inoculated with each bacterium or a mixture of all three species, as described in the PGPR preparation section above, and sown in a randomized block design. To ensure successful bacterial establishment, a further inoculation of 200 ml (10^8^ cfu ml^−1^) of each *Bacillus* formulation was applied to the soil around each treated plant one month after sowing. Controls received an equal volume of sterilized water. There were 20 replicates of each of the five treatments. The site was hand weeded, but no fertilizers or pesticides were applied, since one aim of the experiment was to examine the effects of microbial addition on insect pest infestation levels. After 16 weeks of growth, mature plants were harvested and fresh and dry root and shoot biomass recorded.

### Indian field experiment

This field experiment was carried out at Khandala, India (18°3′N 74°2′E), from March to June, 2014. The experimental plot had a black cotton soil, with 162 kg/ha available nitrogen, 5.6 kg/ha available phosphorus and soil pH of 6.7. Calabrese seeds cv. Imperial (Sakata Seed Corporation, Kanagawa, Japan) from each treatment were inoculated as described in the PGPR preparation section above. The same five treatments as in the UK experiment were used, with 20 replicates of each. These were planted in a randomized complete block design. Seedlings were transplanted 20 d after sowing when they attained 8–10 cm height. A further soil application of each bacterial treatment was applied one month after transplanting.

The plot was irrigated every alternate day, no pesticides and fertilizers were applied throughout the experiment and hand weeding was practiced once every 3 weeks. Plants were harvested 14 weeks after sowing, and fresh root and shoot biomass recorded.

### Data analysis

All analyses were conducted in R 3.4.1. All data reported here pertain to fresh weight; results for dry biomass were extremely similar, and are not presented here to avoid repetition. Inequality in final plant size was examined with a range of measures: the Coefficient of Variation (CV), Gini Coefficient, Gini Mean of Differences and the Lorenz Asymmetry Coefficient, calculated from the production of Lorenz curves. The CV is the simplest to compute and has the advantage that a statistical test can be used to compare CVs^29^. The package ‘zar5’ was used to make these comparisons.

More informative measures of size inequality are the Gini coefficient and Lorenz asymmetry coefficient, both computed from the graphical depiction of inequality, the Lorenz curve. The latter is produced when the cumulative proportion of total biomass (y axis) is plotted against the cumulative percentage of individuals in a population (x axis). If all individuals are identical in size, a diagonal straight line will be obtained, termed the line of equality. If individuals vary in size, a curve will be produced, beneath the line of equality. A comprehensive description of Lorenz curves and the calculation of these statistics is available^30^ and the package ‘ineq’ was used to compute them. Briefly, the Gini coefficient is the ratio of the area enclosed by the Lorenz curve and the line of equality and the total triangular area beneath the line of equality. Meanwhile, the Lorenz asymmetry coefficient is the location of the point where the Lorenz curve has a slope of 1. A coefficient >1 indicates that larger individuals contribute most to the overall inequality, while a value <1 indicates that there is a preponderance of small individuals.

For populations that are non-normal (a common occurrence with plant size), the Gini Mean of Differences (the mean of all pairwise differences) has been suggested as an alternative measure^31^ for measuring inequality and so this was also calculated, using the ‘Hmisc’ package. Confidence intervals for all coefficients were computed using a bootstrap procedure using the ‘boot’ package and non-overlap of intervals deemed to be significant at the 95% level^32^.

## Data Availability

The datasets generated and analysed during the current study are available from the corresponding author on reasonable request.
